# Gintonin Enhances Proliferation, Late Stage Differentiation, and Cell Survival From Endoplasmic Reticulum Stress of Oligodendrocyte Lineage Cells

**DOI:** 10.3389/fphar.2019.01211

**Published:** 2019-10-08

**Authors:** Mohammad Al Mijan, Ji Young Kim, So-Young Moon, Sun-Hye Choi, Seung-Yeol Nah, Hyun-Jeong Yang

**Affiliations:** ^1^Department of Integrative Biosciences, University of Brain Education, Cheonan, South Korea; ^2^Korea Institute of Brain Science, Seoul, South Korea; ^3^Department of Anesthesiology and Pain Medicine, Yonsei University College of Medicine, Seoul, South Korea; ^4^Ginsentology Research Laboratory and Department of Physiology, College of Veterinary Medicine, Konkuk University, Seoul, South Korea

**Keywords:** gintonin, oligodendrocyte, proliferation, differentiation, ER stress, cell protection

## Abstract

Although evidence on myelin diseases is steadily accumulating, effective preventive or therapeutic strategies against them have not been established so far. Ginseng is well known for its beneficial effects on health and diseases; however, detailed studies on ginseng’s effects on myelin-producing oligodendrocytes have not been performed yet. In this study, we investigated the function of gintonin—an active component of ginseng—on the proliferation, differentiation, and survival of oligodendrocyte lineage cells. We performed real-time percutaneous coronary intervention, Western blot, and immunocytochemistry on primary oligodendrocyte precursor cell cultures and *in vitro* myelinating co-cultures. Our results show that gintonin stimulates oligodendrocyte precursor cell proliferation. Gintonin’s effect was inhibited by Ki16425, an antagonist of lysophosphatidic acid 1/3 receptors. Interestingly, with regard to cell differentiation, gintonin facilitated late differentiation of oligodendrocyte development, but not early differentiation. Moreover, it showed protective effects on oligodendrocyte lineage cells against endoplasmic reticulum stress-induced cell death, potentially by modulating unfolded protein responses. Our results suggest that gintonin is a potential therapeutic candidate in the treatment of myelin diseases.

## Introduction

Oligodendrocytes are glial cells that synthesize myelin, a material that wraps around axons and facilitates neuronal conduction in the central nervous system (Hughes and Appel, 2016). Oligodendrocytes are derived from oligodendrocyte precursor cells (OPCs). Expression of transcription factors gradually changes along the developmental stages of oligodendrocyte lineage cells (Dwight E, 2016). During OPC development, certain transcription factors are specifically expressed during the specific developmental stages. For example, Hes5, Id2, Id4, Sox5, and Sox6 are expressed during the OPC stage and Myrf, Zfhx1b, Smad7, and Nkx6-2 are expressed for the later stages (Cahoy et al., 2008). OPCs comprise approximately 5–8% of the glial cell population in adults. In demyelinating conditions, OPCs have the ability to differentiate into myelinating oligodendrocytes to replace the lost myelin (Levine et al., 2001). The number of OPCs is significantly reduced in demyelinated lesions from postmortem patients with multiple sclerosis (Lucchinetti et al., 1999; Chang et al., 2000; Boyd et al., 2013), suggesting abnormal homeostatic control of myelination.

OPCs are highly vulnerable to stress because their metabolic rate and intracellular iron are high, while their concentrations of the antioxidant glutathione are low (Bradl and Lassmann, 2010). Oligodendrocytes produce huge amounts of myelin, and they are hypersensitive to homeostatic alterations such as endoplasmic reticulum (ER) stress (Lin and Popko, 2009). ER stress in myelinating oligodendrocytes plays critical role in causing myelin diseases such as Pelizaeus–Merzbacher disease, Charcot–Marie–Tooth disease, vanishing white matter disease, and multiple sclerosis (Lin and Popko, 2009). Under ER stress conditions, translation is repressed most rapidly (Harding et al., 2000), leading to cell death in actively (re)myelinating oligodendrocytes (Lin and Popko, 2009).

The components of gintonin include lysophosphatidic acids (LPAs), ginseng major latex-like protein 151, and ginseng ribonuclease-like storage protein (Hwang et al., 2012). Gintonin is a high-affinity ligand for mammalian GTP-binding protein-coupled LPA receptors (Hwang et al., 2012). Its functions have been mainly reported in neurons and astrocytes, and those in oligodendrocytes have not been investigated yet.

The pathogenesis of the myelin diseases can derive from various stages of oligodendrocyte development, including proliferation, differentiation, and myelination. In this study, we evaluated whether gintonin can modulate the behavior of oligodendrocyte lineage cells under physiological and pathological conditions. We examined whether gintonin changes OPC proliferation and differentiation during cell development and whether it also affects cell survival and gene expression level under ER stress.

## Materials and Methods

### Mice

All primary cultures were performed in compliance with the relevant laws and institutional guidelines and were approved by the University of Brain Education’s Animal Care and Use Committee (Approval number: 2017-AE-01). Timed pregnant females (day 13.5 of pregnancy) or pups (postnatal day 0–1) of CrljOri : CD1(ICR) mouse line were purchased from ORIENT BIO Inc. (Seongnam, Korea).

### Materials

Six-year-old Korean ginsengs were purchased from local ginseng market and identified by Professor SY Nah, and the specimen (voucher number: NIBRVP0000730014) was deposited at the herbarium of the National Institute of Biological Resources (Herbarium Code: KB, http://sweetgum.nybg.org/science/ih/herbarium-details/?irn=138656). Gintonin was prepared as previously described (Mi Kyung Pyo et al., 2011). Ki16425 was purchased from Cayman Chemicals (Ann Arbor, MI, USA). Tunicamycin was purchased from Sigma-Aldrich (St Louis, MO, USA). Culture medium and reagents were purchased from Invitrogen (Carlsbad, CA, USA).

### Cell Cultures

For OPC cultures, glia mixed culture was prepared in advance. To perform glia mixed cultures, zero to one day old ICR mice were sacrificed for collection of brains. Mouse cortices were separated in L15 on ice and collected into 15 ml falcon tubes, then gently broken down twice with 19xneedles and twice with 21× needles in precooled DMEM/penicillin-streptomycin. Proliferation medium (DMEM F12, 10% fetal bovine serum, 5% horse serum, and 1% penicillin-streptomycin) was added up to 10–15 ml and centrifuged at 1000×*g* for 5 min. The cell pellet was re-suspended in the proliferation medium and transferred to poly-D-lysine (PDL)-coated 75 cm^2^ cell culture flasks. After adding the medium up to 15 ml, cultures were mildly agitated for equivalent distribution of the glial cells. Glial cells were incubated in the proliferation medium at 37°C in a humidified atmosphere with 5% CO_2_. Old medium was replaced with fresh medium every 3 days.

For OPC isolation, on days *in vitro* (DIV) 10 of glia mixed cultures, the medium was aspirated and 10 ml of fresh proliferation medium was added to the culture flask. The flask was vigorously shaken 30 times in a horizontal motion to detach the cells. After observing the OPC detachment under microscope, the medium was collected and centrifuged at 1000×*g* for 5 min. The cell pellet was re-suspended in the proliferation medium for proliferation assay and differentiation medium (DMEM containing 1× B-27 supplement, 1× Glutamax, 1× penicillin–streptomycin, 1% horse serum, 1× sodium pyruvate, 0.34 μg/ml T3, and 0.4 μg/ml T4) for differentiation assays and coculture medium (DMEM containing B-27 supplement, N-2 supplement, 5 μg/ml N-Acetyl-Cysteine, 5 μM forskolin, and penicillin–streptomycin) for myelinating cocultures. Resuspended pellets were incubated on the surface of the petri dishes for 2 min in order to remove astrocytes and transferred carefully for seeding. A number of 4–8 × 10^4^ OPC cells per well were seeded in the 24-well plates.

For cocultures, mouse dorsal root ganglion (DRG) neuronal cultures were separately prepared paralleled with glia mixed cultures. DRGs were dissected out from embryonic 13.5 mouse embryos and dissociated with trypsin and the dissociated neurons were plated 3–4 × 10^4^ cells per slide. Neurons were maintained in neurobasal medium (neurobasal medium containing 1× B-27 supplement, 1× Glutamax, 0.05μg/ml Nerve Growth Factor (NGF), 1× penicillin–streptomycin) with or without FuDR cycle of every two days to induce cell cycle arrest to all proliferating cells. After 2 weeks, NGF was removed from the medium and the neurons were ready for cocultures with OPCs (Yang et al., 2016).

### Lactate Dehydrogenase Cytotoxicity Assay

Cell viability was measured using lactate dehydrogenase (LDH) cytotoxicity detection kit (Takara Bio Inc., Mountain View, CA, USA). 2.7×10^4^ cells suspended in 100 µl of proliferation medium were plated in each well of a 96-well plate and incubated at 37°C and with 5% CO2 in a humidified atmosphere for overnight. Cells were then treated with gintonin along with tunicamycin in fresh medium and incubated overnight. After incubation, the microtiter plate was centrifuged at 250×*g* for 10 min; 70 µl of supernatant was mixed with 70 µl of reaction mixture and incubated for 30 min at RT in darkness. Absorbance was measured at 490 nm and the cell viability was calculated as manufacturer’s instructions.

### Quantitative Real-Time Polymerase Chain Reaction

Total RNA of OPC cultures or DRG/OPC cocultures was extracted using TRI reagent (Sigma-Aldrich, St. Louis, MO, USA) following the manufacturer’s instructions. cDNA was generated using Superscript First-Strand Synthesis System for RT-PCR (Thermo Fisher Scientific). Real-time polymerase chain reaction (PCR) was performed using PowerUp SYBR Green Master Mix (Life Technologies, Austin, TX, USA). All reactions were carried out in triplicate and the expression of each target gene was normalized with GAPDH. Specific primer sets for target genes were used as follows: ID2 (forward, 5′-CCTGCATCACCAGAGACCTG-3′; reverse, 5′-TTCGACATAAGCTCAGAAGGGAA-3′), ID4 (forward, 5′-AGGGTGACAGCATTCTCTGC-3′; reverse, 5′-CCGGTGGCTTGTTTCTCTTA-3′), SOX10 (forward, 5′-AGCCCAGGTGAAGACAGAGA-3′; reverse, 5′-AGTCAAACTGGGGTCGTGAG-3′), Myrf (forward, 5′-TGGCAACTTCACCTACCACA-3′; reverse, 5′-GTGGAACCTCTGCAAAAAGC-3′), myelin basic protein (MBP) (forward, 5′-CCAGAGCGGCTGTCTCTTCC-3′; reverse, 5′-CATCCTTGACTCCATCGGGCGC-3′), GAPDH (forward, 5′-GGTCGGTGTGAACGGATTTG-3′; reverse, 5′-TCGTTGATGGCAACAATCTCCACT-|3′), Olig1 (forward, 5′-GCTCGCCCAGGTGTTTTGT-3′; reverse, 5′-GCATGGAACGTGGTTGGAAT-3′), 2', 3'-cyclic nucleotide 3'-phosphodiesterase (CNP) (forward, 5′-GTTCTGAGACCCTCCGAAAA-3′; reverse, 5′-CCTTGGGTTCATCTCCAGAA-3′), proteolipid protein (PLP) (forward, 5′-GGTACAGAAAAGCTAATTGAGACC-3′; reverse, 5′-GATGACATACTGGAAAGCATGA-3′), BIP (forward, 5′-ACTCCGGCGTGAGGTAGAAA-3′; reverse, 5′-AGAGCGGAACAGGTCCATGT-3′), GRP94 (forward, 5′-TGGGTCAAGCAGAAAGGAG-3′; reverse, 5′-TCTCTGTTGCTTCCCGACTT-3′), CHOP (forward, 5′-CCACCACACCTGAAAGCAGAA-3′; reverse, 5′-AGGTGCCCCCAATTTCATCT-3′), and sXBP-1 (forward, 5′-AAGAACACGCTTGGGAATGG-3′; reverse, 5′-CTGCACCTGCTGCGGAC-3′).

### Immunoblot Analysis

Western blot analysis was carried out to assess the expression of the desired proteins. Cells were harvested and directly lysed with sample buffer. Samples were separated by sodium dodecyl sulfate polyacrylamide gel electrophoresis and transferred to polyvinylidene difluoride (PVDF), membranes (Bio-Rad Laboratories Inc., Hercules, USA). After blocking with 5% bovine serum albumin (RMBIO, Missoula, MT, USA)/tris buffered saline with tween 20 (TBST), the membranes were incubated overnight at 4°C with following primary antibodies: rat anti MBP (1:5000, Merck, Darmstadt, Germany), rabbit anti XBP-1s (1:1000, D2C1F, Cell Signaling Technology, Inc., Danvers, MA, USA), extracellular signal-regulated protein Kinase (ERK1/2, 1:20,000, sigma), phospho-p44/42 (1:2,000, Cell Signaling Technology, Inc.), and beta-actin (1:5000, Abcam, Cambridge, MA, USA) antibodies. The membranes were probed with following secondary antibodies: horseradish peroxidase conjugate goat anti-rabbit IgG (1:5,000, Life Technologies, Frederick, MD, USA) or goat anti-rat IgG (1:10,000, Jackson ImmunoResearch Laboratories, Inc., West Groove, PA, USA) for 1 h at room temperature. Images were obtained using an Amersham Imager 600 (GE Healthcare, Pittsburgh, PA, USA) and quantified with Image J Software.

### Immunofluorescence

For immunofluorescence, cells were fixed with 4% paraformaldehyde/phosphate-buffered saline (PBS) for 15 min at room temperature and blocked with PBS containing 5% normal goat serum, 0.5% Triton X-100 for 1 h, then stained with primary antibodies for overnight at 4°C. Cells were washed with PBS, incubated for 1 h with secondary antibodies, washed in PBS and mounted with Vectashield mounting solution (Vector Laboratories, Burlingame, CA, USA). Following primary antibodies were used for immunocytochemistry: rabbit antibodies to Olig 2 (1:500, AB9610, Millipore), ki67 (1:500, SP6, Cell Marque), rat antibody to MBP (1:300, MAB386, Chemicon), and hybridoma supernatants of mouse antibody to O4 (1:10). Secondary antibodies were obtained from Jackson Immunoresearch and Invitrogen. For O4 staining, cells were lively stained for 45 min in the incubator before fixation. Images were captured with a LSM700 confocal microscope (Carl Zeiss) and analyzed with Image J.

### Statistical Analyses

All data were represented as mean ± S.E.M. Statistical analyses were performed using unpaired *t*-test, one- or two-way analysis of variance (ANOVA) with Tukey, Dunnett, or Bonferroni *post hoc* tests where appropriate. Differences were significant at *p* < 0.05.

## Results

### Gintonin Enhances the Proliferation of Oligodendrocyte Lineage Cells

**Figure 1:**
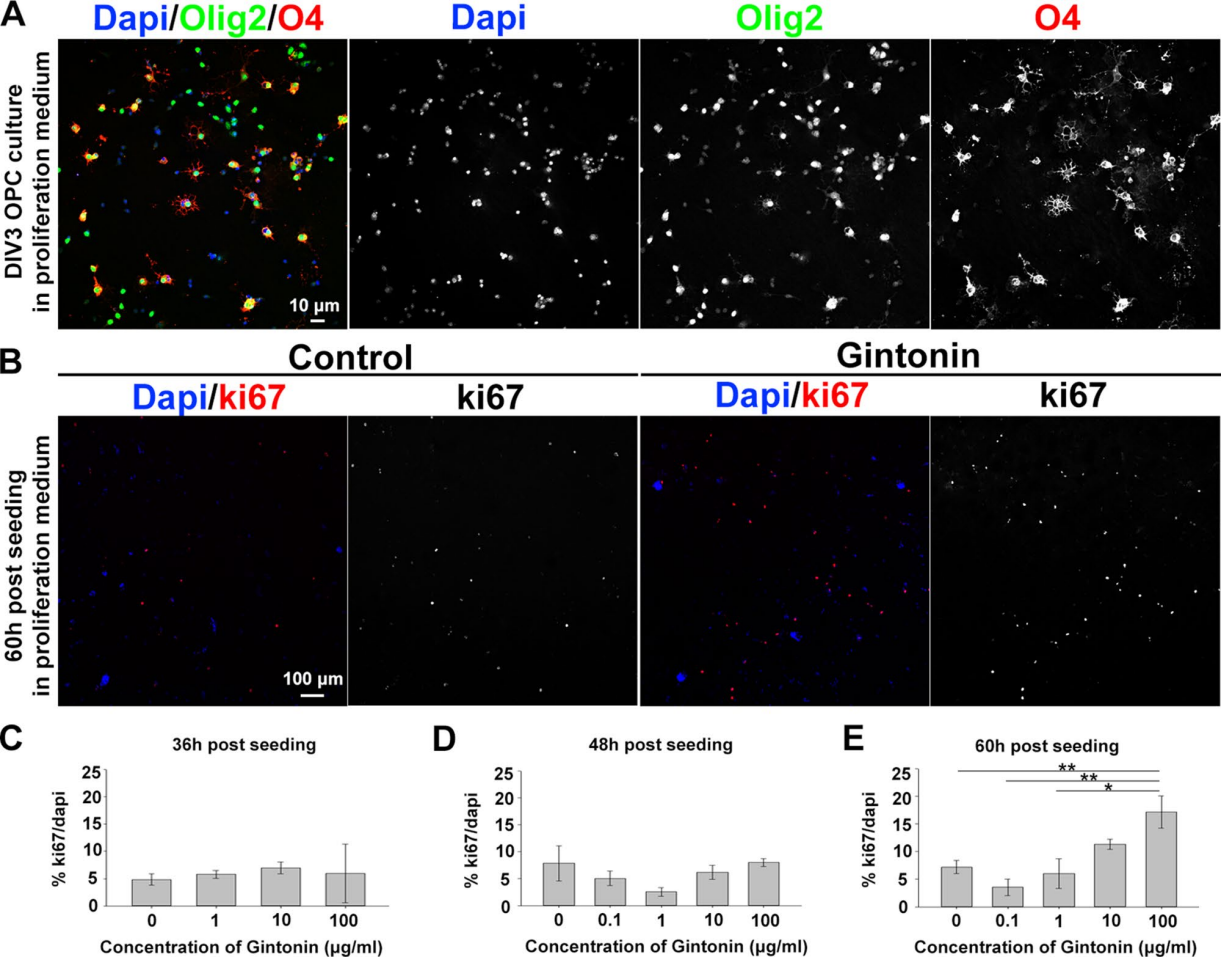
Gintonin enhances proliferation of oligodendrocyte lineage cells in dose- and time-dependent manner. **(A)** Representative image of OPC culture stainings to DAPI, olig2, and O4 in proliferation medium at DIV3 is shown. **(B**–**E)** OPCs were cultured in proliferation medium and incubated with gintonin of 0 to 100 µg/ml for 36 **(C)**, 48 **(D)**, and 60 h **(E)**. After the indicated time period, cells were fixed for staining of Ki67 and DAPI for proliferation analysis. Representative pictures at 60 h post seeding are shown **(B)**. Significant increase was found in proliferation ratio of samples at 60 h post seeding **(E)** (*p < 0.05, **p < 0.01, one way ANOVA, Bonferroni post-test, N = 3–8). Bars represent mean ± S.E.M. Scale bars, 10 µm **(A)**, 100 µm **(B)**.

To understand whether gintonin affects the initial step of oligodendrocyte development, we investigated OPC proliferation by gintonin treatment at different concentrations and at various time points in the proliferation medium that was devoid of differentiation-inducing hormones such as T3 and T4. OPCs were isolated from mixed glial cultures at DIV10 by shaking, seeded on PDL-coated coverslips, and allowed to adhere. The purity of OPC cultures was investigated by calculating Olig2+cell number/DAPI+cell number (%) at DIV3 in proliferation medium. The OPC purity in our system was 91.0 ± 1.6% (mean ± S.E.M.) (**Figure 1A**). The medium was changed with fresh medium containing different concentrations of gintonin: 0, 0.1, 1, 10, and 100 μg/ml. After 36, 48, and 60 h of seeding, cells were fixed for immunocytochemistry using an antibody against proliferation marker Ki67 and DAPI (**Figure 1B**). There were no significant changes in the proliferation ratio (% Ki67/DAPI) until 48 h after seeding (**Figures 1C, D**, **Supplementary Figures S1A–D**); however, there was a concentration-dependent increase in the proliferation ratio at 60 h after seeding; 3.5, 6.0, 11.3, and 17.2% for 0.1, 1, 10, and 100 μg/ml, respectively (**Figures 1B, E**, **Supplementary Figures S1E, F**). There were no significant changes in the proliferation of oligodendrocyte lineage cells treated with gintonin dissolved in the differentiation medium containing T3 and T4 at the same time points (**Supplementary Figures S1G–I**).

**Figure 2:**
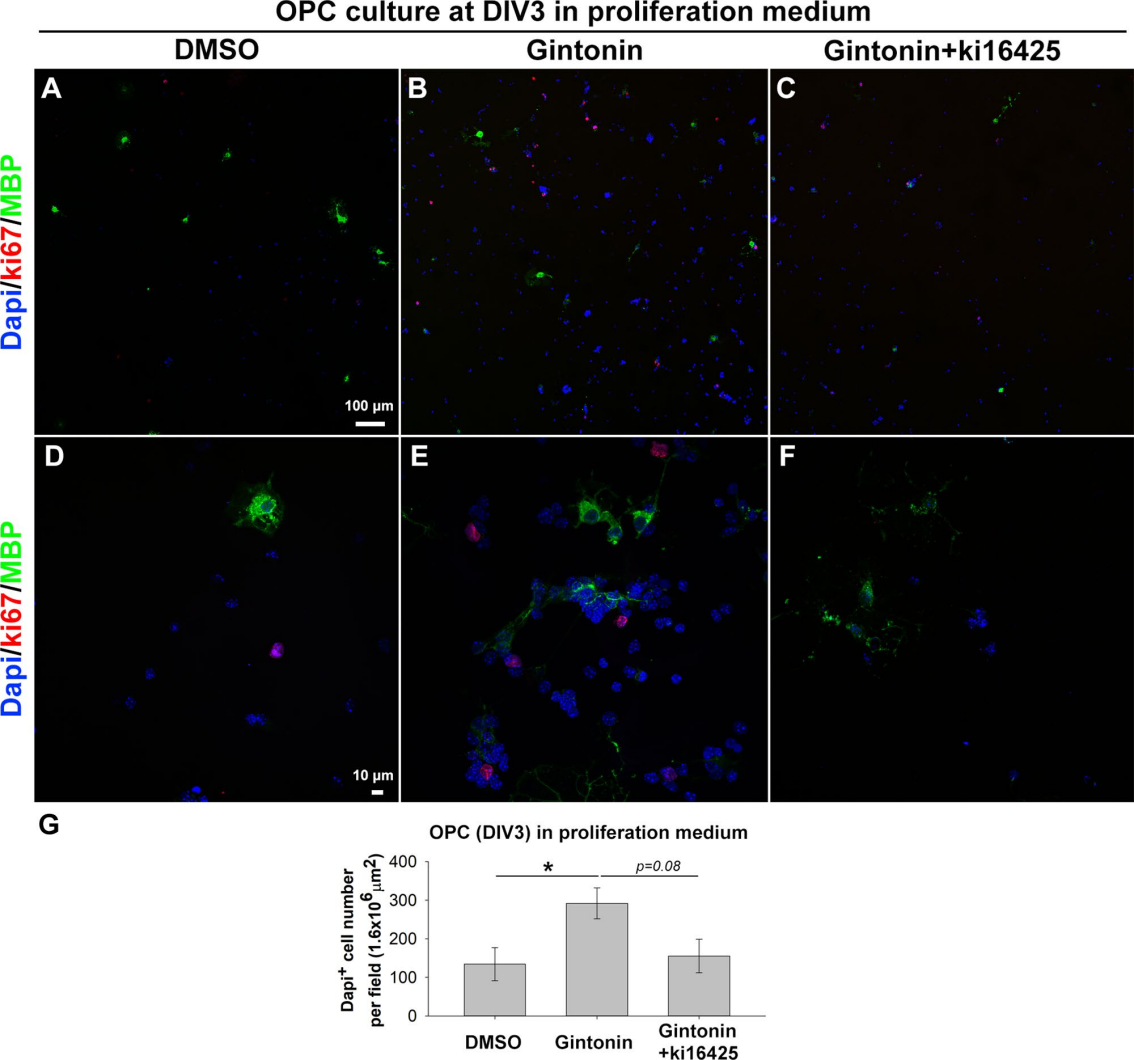
Gintonin-induced proliferation is inhibited by Ki16425, a LPA 1/3 receptor antagonist. Pure OPC s were treated as indicated, with 10 µg/ml gintonin or 1 µM Ki16425 in the proliferation medium during DIV1–3 and fixed for proliferation analysis. **(A**–**F)** Cells were visualized by staining with DAPI (blue), antibodies to Ki67 (red), and MBP (green). Scale bars＝100 **(A**–**C)** and 10 µm **(D**–**F)**. **(G)** DAPI^+^ cell number was automatically counted per field by image J. Cell number was significantly increased by gintonin treatment (*p < 0.05) and showed a tendency of downregulation by Ki16425 treatment (*p* = 0.08, one way ANOVA, Bonferroni post-test, N = 6–13). Bars represent mean ± S.E.M.

Previous studies have shown that gintonin containing LPAs acts through LPA receptors (Hwang et al., 2012; Choi et al., 2019). As LPA1 and LPA3 expression on oligodendrocytes has been reported (Allard et al., 1998; Weiner et al., 1998; Yu et al., 2004), we made use of Ki16425, an antagonist of LPA1 and LPA3 receptors to investigate whether the increase of cell number was mediated by LPA1/3 receptors. OPCs were treated with the control proliferation medium, 10 μg/ml gintonin with or without 1 μM Ki16425 during DIV1–3. The inhibitor was added 30 min before the incubation with gintonin, to block LPA1/3 receptors. Treating with Ki16425 did not affect the OPC proliferation (**Supplementary Figure 2**) throughout this research, indicating that Ki16425 produced no cytotoxic effect on OPCs, which was consistent with a previous report (Nakajima et al., 2018). At DIV3, cells were fixed for proliferation analysis. Results from proliferation assay showed that the oligodendrocyte lineage cell number was significantly increased by gintonin treatment, while this increase was returned to the similar level of the control group by Ki16425 (**Figure 2**), suggesting that the increase in cell proliferation by gintonin was mediated by LPA 1/3 receptors in OPCs.

### Gintonin Affects Late-Stage Differentiation of Oligodendrocyte Lineage Cells But Not Early-Stage Differentiation

**Figure 3:**
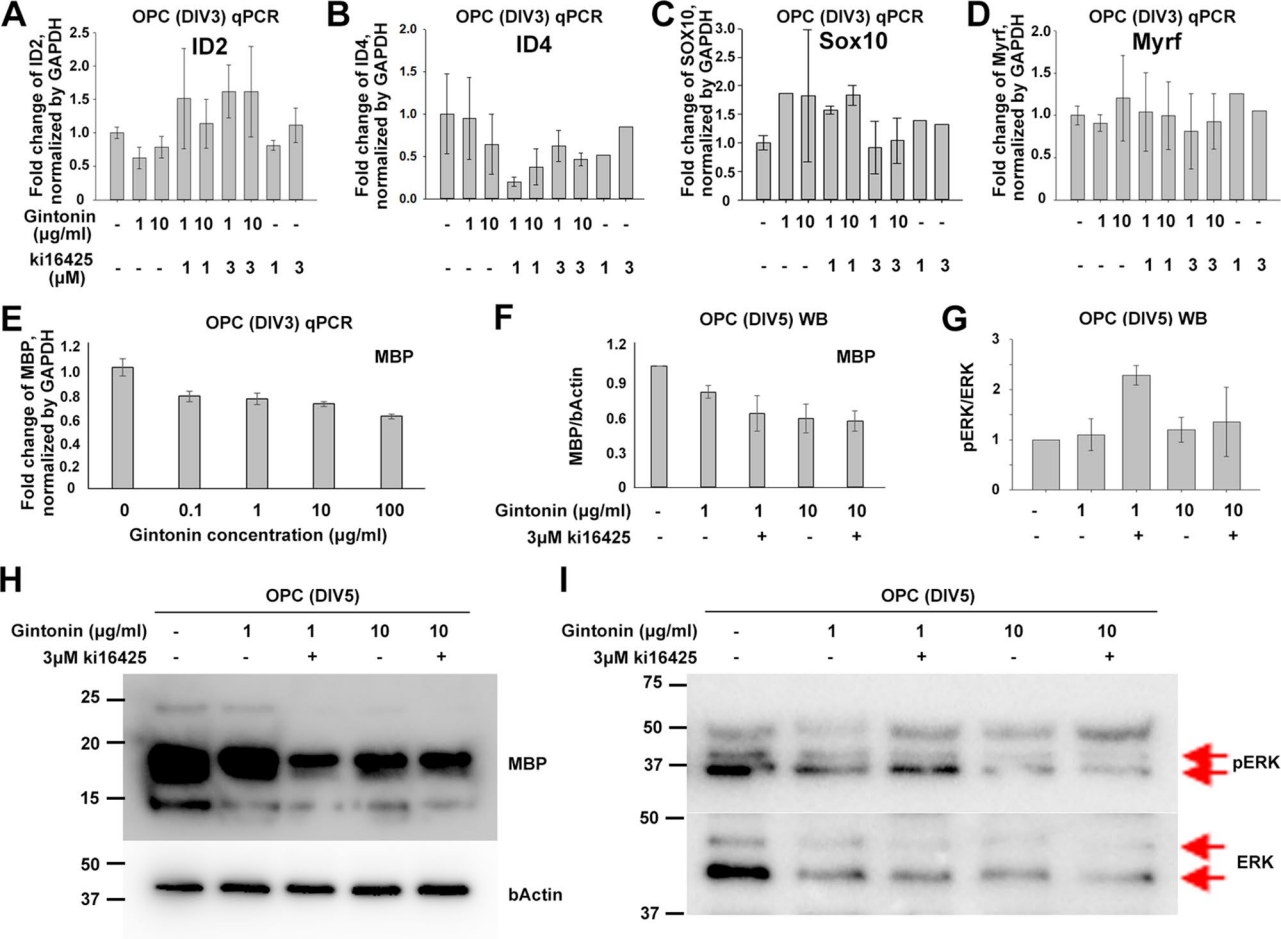
Gintonin does not change the expression of transcription factors at early stage of oligodendrocyte development. For differentiation analysis, isolated OPC s were treated with gintonin or Ki16425 or both of them as indicated during DIV1–3 **(A**–**E)** or DIV1–5 **(F**–**I)**. Medium was changed every two days. **(A**–**E)** At DIV3, RNA expression of inhibitory **(A**, **B)**, facilitatory **(C**, **D)** transcription factors of oligodendrocyte differentiation and myelin gene expression **(E)** were investigated on OPC cultures by real-time PCR. **(F**–**I)** At DIV5, protein expression levels of MBP **(F**, **H)** and phospho ERK (pERK) **(G**, **I)** were investigated on OPC cultures by Western blot. Protein bands of ERK and pERK are indicated by red arrows **(I)**. No significant changes were found by gintonin treatment in the real-time PCR or Western blot analysis. Bars represent mean ± S.E.M.

To see whether gintonin also affects the differentiation of oligodendrocyte lineage cells and whether it is occurred through LPA 1/3 receptors-mediated mechanisms or not, OPCs were suspended in the differentiating medium containing T3 and T4 on the day of the seeding and incubated with reagent-containing differentiation medium during DIV1–3, followed by the cell collection at DIV3 for quantitative PCR (qPCR) on inhibitory (ID2, ID4) and facilitatory (SOX10, Myrf) transcription factors of differentiation (**Figures 3A–D**). The results showed that there were no significant changes in the expression of differentiation-regulating transcription factors by gintonin and Ki16425, although there were slight reduction in ID2 (**Figure 3A**), ID4 (**Figure 3B**) expression and mild increase in SOX10 (**Figure 3C**) and Myrf (**Figure 3D**) expression by gintonin treatment.

As MBP is the representative marker of mature oligodendrocytes, we investigated MBP expression depending on gintonin concentration (0.1, 1, 10, and 100 μg/ml) by qPCR at DIV3 when a mixture of oligodendrocyte lineage cells of various developmental stages coexist. However, we found no significant changes in MBP expression in relation to the concentration of gintonin at DIV3 (**Figure 3E**). To see whether the MBP protein expression was modulated by gintonin treatment, we treated cells with either gintonin (1, 10 μg/ml) and gintonin with LPA1/3 receptor antagonist (3 μM Ki16425) from DIV1 and collected them at DIV5 for Western blot with medium change of every other day. Consistent with the qPCR results, no significant changes were found in MBP protein expression by gintonin treatment (1, 10 μg/ml) as well as by LPA 1/3 receptor antagonist (Ki16425) (**Figures 3F, H**, **Supplementary Figure S3**), unlike proliferating OPCs (**Figure 2**).

ERK signaling is one of the main signal transduction pathways in oligodendrocytes (Guardiola-Diaz et al., 2012). To know whether gintonin-mediated changes occurred through ERK pathway, we prepared same samples as above and performed immunoblot for pERK and ERK antibodies (**Figures 3G, I**, **Supplementary Figure S4**). No significant changes by gintonin treatment in ERK phosphorylation were observed. Interestingly, ERK phosphorylation was significantly increased by 3 μM Ki16425 together with 1 μg/ml gintonin but not with 10 μg/ml gintonin. This suggests that LPA1/3 inhibition activates ERK phosphorylation but prolonged incubation with high concentrations (10 μg/ml) of gintonin might reverse ERK phosphorylation-related signaling.

**Figure 4:**
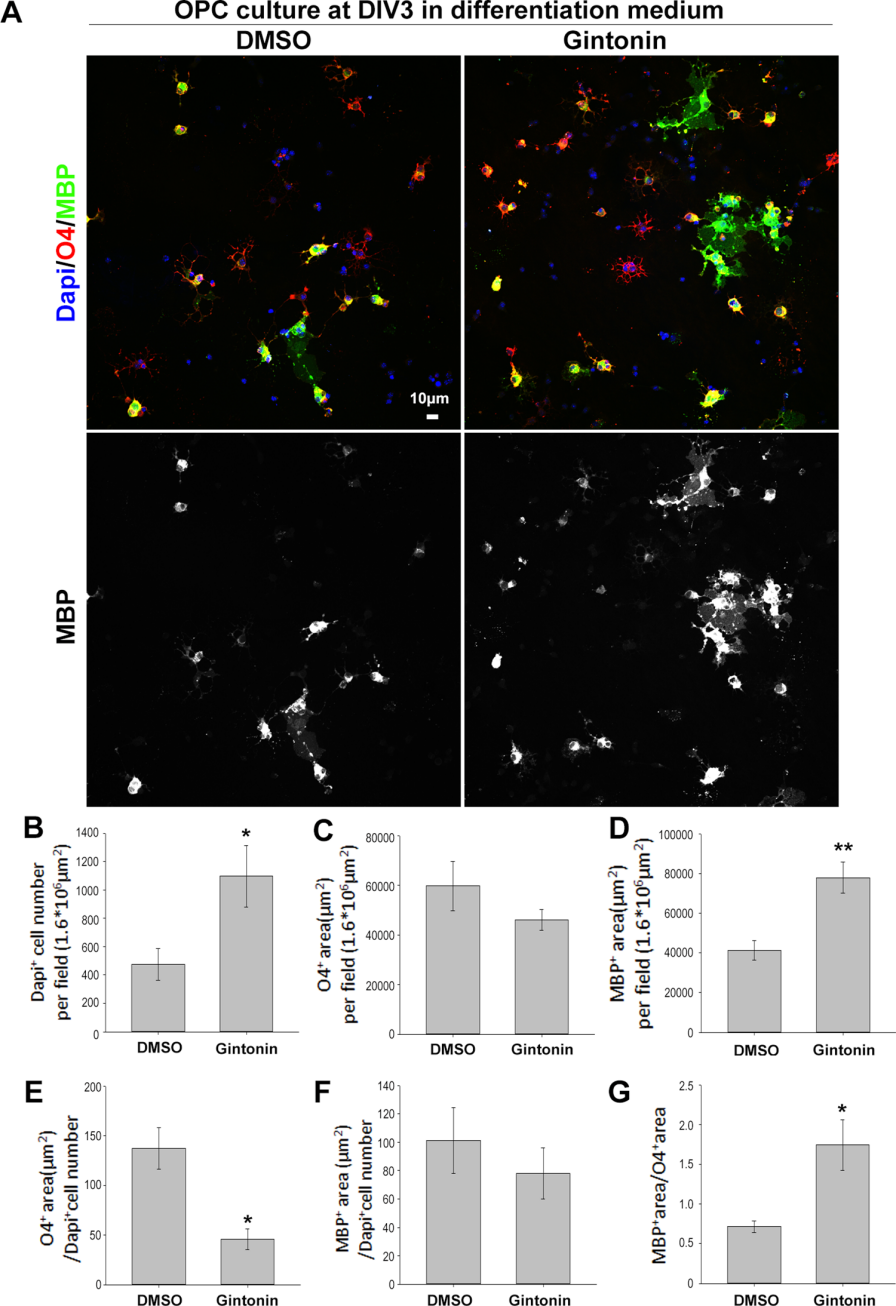
Gintonin facilitates the late stage differentiation of oligodendrocytes. **(A)** For stage-specific differentiation analysis, pure OPC cultures treated with gintonin (10 µg/ml) or DMSO from DIV1 were lively stained at DIV3 with O4 antibodies (a marker for oligodendrocytes from immature stage, red), then allowed to fix for MBP (a mature oligodendrocyte marker, green) and DAPI (a nucleic marker, blue) staining. Scale bar＝10 µm. **(B)** In differentiation medium, DAPI+ cell number per field was significantly increased by gintonin treatment (*p < 0.05). **(C)** O4+ area per field by gintonin treatment. **(D)** MBP+ area per field was significantly enhanced by gintonin (**p < 0.01). **(E)** O4+ area per DAPI+ cell number (*p < 0.05). **(F)** MBP+ area per DAPI+ cell number. **(G)** MBP+ area per O4+ area was calculated as late differentiation ratio and was significantly increased by gintonin treatment (*p < 0.05). Bars represent mean ± S.E.M. Two-tailed unpaired Student’s *t*-test, N = 3–4.

Oligodendrocyte lineage cells go through several stages from proliferating precursor cells to mature myelinating oligodendrocytes. Various factors affect each stage, regulating myelin homeostasis in the brain (Bergles and Richardson, 2016). To evaluate whether there were changes in the developmental stages, which are mainly morphological and hard to identify by sample measurements using techniques such as real-time PCR or Western blot, we performed immunocytochemistry using stage-specific markers of O4 (for immature and mature stages) and MBP (for mature stage) (**Figure 4A**). Pure OPC cultures were treated with dimethyl sulfoxide (DMSO) or 10 μg/ml gintonin in differentiation medium from DIV1 and fixed on DIV3 for analysis. Consistent with the proliferation results (**Figures 1** and **2**),gintonin was found to cause higher cell proliferation even in differentiation medium (**Figure 4B**). O4+ area per field was not significantly changed by gintonin treatment (**Figure 4C**), resulting in lower O4+ area per cell (**Figure 4E**), suggesting that gintonin does not affect early oligodendrocyte differentiation. Interestingly, MBP+ area per field was significantly higher in gintonin treatment compared to the control (**Figure 4D**). As DAPI+ cell number was high in gintonin-treated cultures (**Figure 4B**), MBP+ area per cell was not significantly different (**Figure 4F**). However, MBP+ area/O4+ area, which indicates the ratio of mature oligodendrocytes among immature–mature oligodendrocytes, showed higher value in gintonin-treated cultures compared to DMSO controls (**Figure 4G**). This suggests that gintonin may facilitate the differentiation in the late stages of oligodendrocyte development. Moreover, the effects of gintonin on late stage differentiation was not significantly related with the concentration of gintonin (**Supplementary Figure 5**), distinct with its effects on OPC proliferation (**Figure 1E**), implying that gintonin may work through a different mechanism in each condition.

### Gintonin Protects Oligodendrocyte Lineage Cells From ER Stress-Induced Cytotoxicity and Drives Recovery of Oligodendocyte-Differentiation Gene Expression

**Figure 5:**
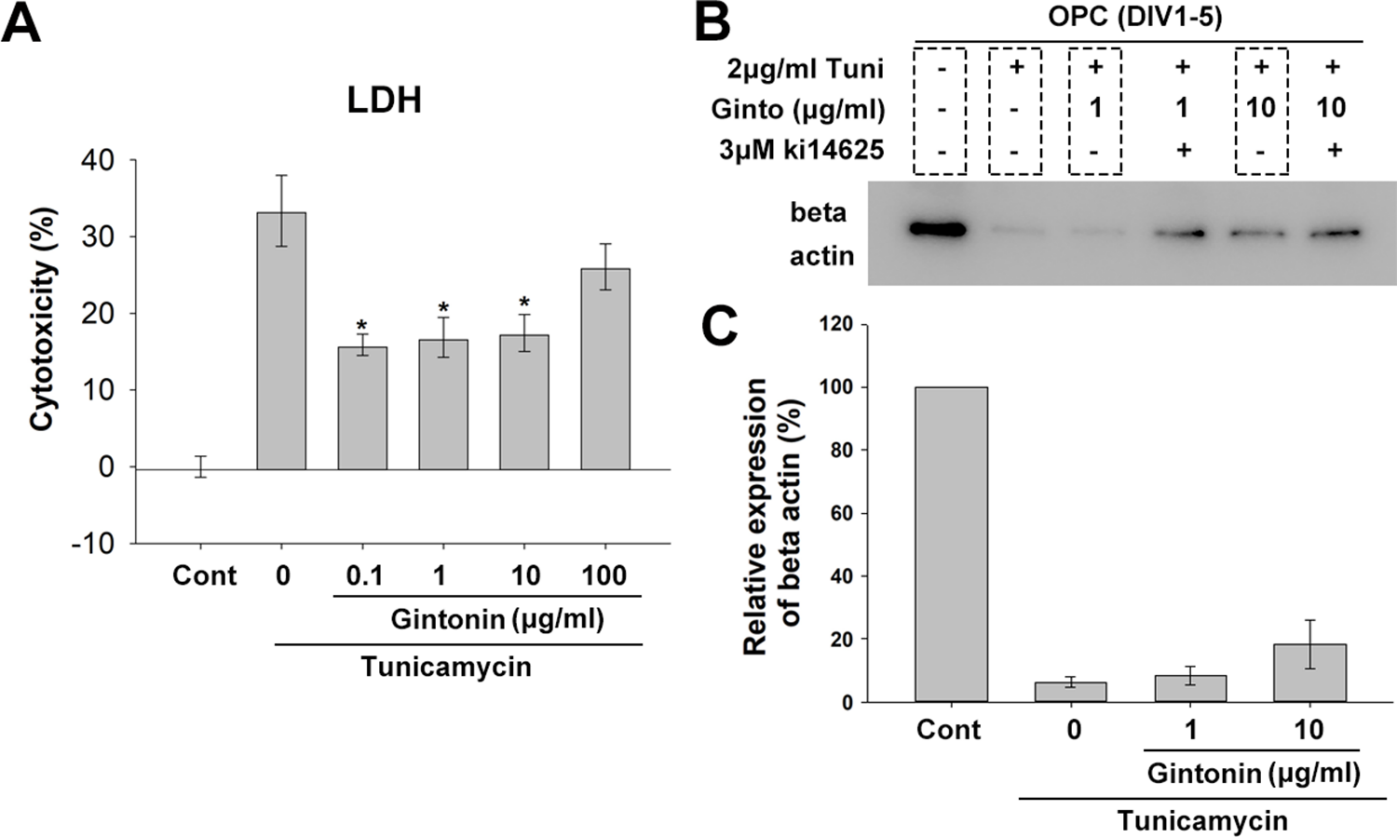
Gintonin protects oligodendrocyte lineage cells against ER stress-induced cytotoxicity. **(A)** Oligodendrocyte precursor cell (OPC) cultures were treated with gintonin of various concentrations (0.1, 1, 10, 100 µg/ml) under 5 µg/ml tunicamycin during DIV 1–2 and LDH assay was performed at DIV2 (*p < 0.05, N = 3). **(B**, **C)** OPC cultures were exposed to long term ER stress induced by tunicamycin during DIV1–5 with or without gintonin or LPA inhibitors of indicated concentrations and submitted to Western blot analysis **(B)** and the expression quantification of beta actin was performed **(C)**. Bars represent mean ± S.E.M.

Under ER stress, which is a critical factor in causing myelin diseases (Lucchinetti et al., 1999), translation in oligodendrocytes is repressed (Harding et al., 2000), which leads the cell death (Lin and Popko, 2009). Gintonin has been reported to protect neurons against death and act as an anti-inflammatory and anti-oxidant agent (Choi et al., 2018). To know whether gintonin plays a role in protecting oligodendrocytes under ER stress, we next incubated OPCs with gintonin at various concentrations (0, 0.1, 1, 10, 100 μg/ml) under overnight ER stress induced by 5 μg/ml tunicamycin treatment during DIV1–2, and measured the cytotoxicity using LDH assays. Tunicamycin increased the expression of ER stress marker BIP in our system (**Supplementary Figure 6**). Tunicamycin-induced cytotoxicity was significantly reduced by gintonin at 0.1, 1, and 10 μg/ml (**Figure 5A**). This suggests that gintonin of 0.1–10 μg/ml can protect oligodendrocytes under ER stress.

Under ER stress, translation is repressed, resulting in low protein expression. To evaluate whether gintonin changes this phenotype, we incubated OPCs with tunicamycin for inducing ER stress during DIV1–5 while the medium was changed every 2 days. Protein expression was measured with β-actin. As expected, beta actin expression was dramatically reduced by tunicamycin treatment (**Figures 5B, C**, **Supplementary Figure S7**). Interestingly, gintonin dose-dependently increased β-actin expression (**Figures 5B, C**), suggesting that gintonin might release the repression of gene expression under ER stress. Moreover, we found Ki16425, which we initially used as an inhibitor of gintonin strongly alleviated the repression, resulting in higher β-actin expression. This suggests that inhibition of LPA1/3 by Ki16425 may also facilitate the gene expression under ER stress.

**Figure 6:**
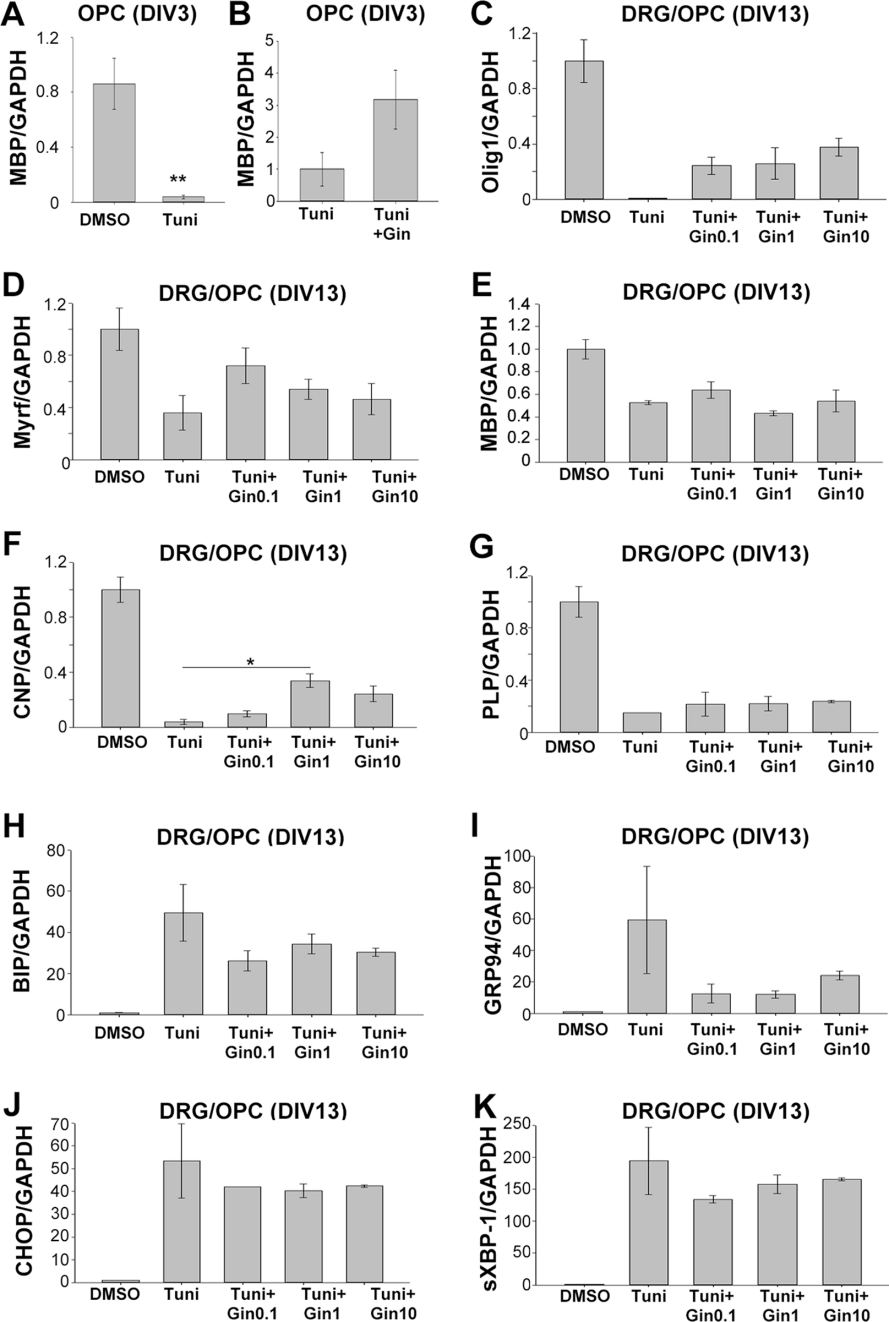
Gintonin reverses the repressed expression of myelin forming-facilitatory genes under ER stress, potentially by modulating UPR responses. Oligodendrocyte precursor cell (OPC) cultures **(A** and **B)** or DRG neurons/OPC myelinating cocultures **(C**–**K)** were treated with gintonin of the indicated concentrations from DIV1 and ER stress was induced by tunicamycin at the final overnight before the collection of total RNA. At DIV3 of OPC, the expression of MBP was investigated **(A** and **B)**. **p < 0.01, Two-tailed unpaired Student’s *t*-test, N = 3. At DIV 13 of DRG/OPC cocultures, the expression of myelin forming-facilitatory genes such as Olig1 **(C)**, Myrf **(D)**, MBP **(E)**, CNP **(F)**, and PLP **(G)** as well as unfolded protein response-related genes such as BIP **(H)**, GRP94 **(I)**, CHOP **(J)**, sXBP-1**(K)** were examined. *p < 0.05, One way ANOVA, Tukey post-test, N = 3. Bars represent mean ± S.E.M.

As gintonin subdued the repression of gene expression under ER stress, we wondered whether it was also applicable to myelin-related gene expression. We investigated myelin-related gene expression in pure OPC culture at DIV3 as well as myelinating DRG/OPC coculture at DIV13. At DIV3 of OPC culture, 2 μg/ml tunicamycin significantly reduced MBP expression (**Figure 6A**). Interestingly, the expression was reversed by 1 μg/ml gintonin in the same condition (**Figure 6B**).

As OPC cultures at DIV3 contain a mixed population of premature and mature oligodendrocytes, we wondered whether gintonin affects the expression of mature oligodendrocytes under ER stress in the myelinating cultures. To find the answer, OPCs were seeded on dissociated DRG neuronal cultures and the co-cultures were incubated with gintonin at various concentrations (0, 0.1, 1, 10 μg/ml). The cultures were treated with 5 μg/ml tunicamycin overnight during DIV12–13, and the samples were collected at DIV13 for real-time PCR against Olig1 (transcription factor for myelinating oligodendrocytes and their progenitors), Myrf (transcription factor for oligodendrocyte differentiation and myelination), MBP, PLP (compact myelin genes), and CNP (non-compact myelin gene). The expression of myelin-related genes were dramatically suppressed by tunicamycin treatment (**Figures 6C–G**). However, gintonin treatment reversed the repression of gene expression under ER stress, resulting in increased Olig1 (**Figure 6C**), Myrf (**Figure 6D**), and CNP (**Figure 6F**) gene expression, as well as mild alterations in MBP (**Figure 6E**) and PLP (**Figure 6G**) gene expression. The expression of ER stress markers such as BIP, GRP94, CHOP, and sXBP-1 was increased by tunicamycin, whereas their expression was mildly reduced by gintonin treatment (**Figures 6H–K**). Current results suggest that gintonin protects oligodendrocyte lineage cells from ER stress-induced cytotoxicity and enhances the recovery of oligodendrocyte-differentiation gene expression under ER stress.

## Discussion

Gintonin contains multiple components. These include phospholipid derivatives such as LPA C18:2 (434 nmol/g weight ginseng), LPA C16:0 (49 nmol/g weight ginseng), and other LPAs (below the sensitivity limit); proteins such as ginseng major latex-like protein and ribonuclease-like storage protein (Hwang et al., 2012); fatty acids such as linoleic, palmitic, oleic, and stearic acids; and carbohydrates such as glucose and glucosamine (Mi Kyung Pyo et al., 2011). Gintonin activates LPA receptor-mediated calcium signaling in B103 rat neuroblastoma cells (Hwang et al., 2012) and stimulates human umbilical vascular endothelial cell proliferation in a concentration-dependent manner *via* LPA1/3 receptor activation (Hwang et al., 2016).

OPCs highly express LPA1, LPA4, LPA6, and LPA8 (also known as P2Y10) (Nakajima et al., 2018). In primary mouse OPC cultures, LPA 18:1 or analog of cyclic phosphatidic acid, whose structure is similar to that of LPA, stimulated OPC proliferation but did not change expression of MBP or CNP at DIV3 in differentiation conditions without growth factor (Nakajima et al., 2018).These findings are consistent with our results of proliferation (**Figure 2G**) and MBP expression (**Figures 3E, F**). Inhibition of LPA1 and LPA3 by Ki16425 abolished the analog of cyclic phosphatidic acid-induced OPC proliferation (Nakajima et al., 2018). This is also in accordance with our results, where gintonin-induced OPC proliferation was inhibited by Ki16425 (**Figure 2G**). There, our results suggest that gintonin increases the OPC proliferation through a LPA1/3 receptor-mediated mechanism.

Some of the transcription factors, such as Olig2 and Sox10, are expressed throughout all the developmental stages, participating in different regulations at each stage (Emery and Lu, 2015). Olig2 functions in specification to the oligodendrocyte lineage cells in neural precursor cells, and later plays a role in initiating the myelination cascade program (Emery and Lu, 2015). SOX10 plays important roles in maintaining OPC populations during developmental stage (Finzsch et al., 2008), and it is a major determinant in the terminal differentiation of oligodendrocytes by interacting with several other proteins (Elbaz and Popko, 2019). Previous studies have reported that LPAs also function in two different developmental stages: OPC proliferation (Nakajima et al., 2018) and differentiation (Wheeler et al., 2015). LPA stimulates OPC proliferation during the precursor stage (Nakajima et al., 2018), whereas it stimulates histone deacetylase 1/2 activation during the differentiation stages; the latter represses the transcription inhibitors for oligodendrocyte differentiation, facilitating oligodendrocyte differentiation (Wheeler et al., 2015). We found that gintonin stimulates OPC proliferation (**Figures 1** and **2**) as well as late-stage of oligodendrocyte differentiation (**Figures 3** and **4**). Current results of gintonin containing LPAs for its active components suggest that it functions in two different developmental stages of oligodendrocytes: the precursor stage and the transition stage from immature to mature oligodendrocytes.

However, while gintonin showed dose-dependent changes in OPC proliferation (**Figure 1E**), it did not show consistent dose-dependent changes in differentiated oligodendrocytes (**Supplementary Figure 5**) as well as ER stress-induced conditions (**Figures 5** and **6**). Gintonin is the complex of multiple components (Mi Kyung Pyo et al., 2011; Hwang et al., 2012). Gintonin seems to work through LPA receptors on OPCs by LPA1/3 (**Figure 2G**). As OPCs express enough LPA receptors which may be activated by input LPAs (Nakajima et al., 2018), OPC proliferation seems to present dose-dependent changes within the used dose. Gene expression dramatically changes when OPCs are differentiated into mature oligodendrocytes (Emery and Lu, 2015). We did not see consistent gintonin dose-dependent changes in differentiated oligodendrocytes (**Supplementary Figure 5**) as well as in ER stress environments (**Figures 5** and **6**). This suggests that other components of gintonin in addition to LPA1/3 may also function in mature oligodendrocytes or stress condition. Moreover, this indicates that the expression or activity of the receptor against the specific components may reach threshold in the dose used in the experiments, resulting in not showing dose-dependent alterations.

To maintain myelin structure, oligodendrocytes produce huge amounts of proteins and lipid-rich membranes, which make them susceptible to ER stress. The unfolded protein response (UPR), which is an initial cell response to ER stress, is cell protective; however, prolonged stress lead to apoptotic cell death. In demyelinating diseases including multiple sclerosis, vanishing white matter disease, Charcot–Marie–Tooth disease, and Pelizaeus–Mezbarcher disease, robust evidence showing that ER stress contributes to the pathology is available (Clayton and Popko, 2016). Alterations in the UPR response by inhibiting GADD34 function, such as by using guanabenz, protected cells against ER stress (Tsaytler et al., 2011). GRP94, BiP, ATF4, CHOP expression induced by tunicamycin treatment was downregulated by guanabenz, confirming protection against cell death (Tsaytler et al., 2011). We have found that gintonin increased gene expression, which was repressed during tunicamycin-induced ER stress (**Figures 5** and **6**). Under ER stress in the myelinating co-cultures, facilitatory gene expression for myelin formation was increased (**Figures 6C–G**), while ER stress marker expression was relatively reduced (**Figures 6H–K**) by gintonin treatment. This effect potentially contributed to protect OPCs from ER stress–induced cell death (**Figure 5A**), suggesting cell-protective effects of gintonin under ER stress. Our study indicates that gintonin may affect protein expression by modulating UPR under ER stress and prevent ER stress-induced cell deaths. This suggests that gintonin or gintonin-derived components can be utilized for myelin maintenance or protection in the demyelinating disorders. Therefore, it is meaningful to investigate the effects of gintonin on myelin homeostasis in demyelination animal models such as lysolecithin or cuprizone models for future studies.

## Conclusion

This is the first detailed study of the effects of gintonin on oligodendrocyte lineage cells. Our results show that gintonin increases proliferation of OPCs *via* LPA1/3 receptors, facilitates late-stage differentiation, and protects oligodendrocyte lineage cells from cell death under ER stress by modulating the UPR response. Our findings warrant further studies to identify the detailed molecular mechanisms underlying gintonin’s effects on oligodendrocyte lineage cells and to investigate its *in vivo* functions using myelin-disease animal models for further therapeutic applications in myelin diseases.

## Data Availability Statement

The raw data supporting the conclusions of this manuscript will be made available by the authors, without undue reservation, to any qualified researcher.

## Ethics Statement

The animal study was reviewed and approved by University of Brain Education’s Animal Care and Use Committee.

## Author Contributions

MM, JK, S-YM, S-HC, and H-JY performed and analyzed experiments; and S-YN and H-JY designed and supervised the research and H-JY wrote the manuscript.

## Funding

This work was supported by Basic Science Research Program through the National Research Foundation of Korea (NRF) funded by the Ministry of Education (2017R1D1A3B03027875) and (NRF 2016M3C7A1913894); University of Brain Education (2017–03); and a faculty research grant of Yonsei University College of Medicine for (6–2014–0004). The funder played no role in the design and conduct of the study; collection, management, analysis, and interpretation of the data; preparation, review, or approval of the manuscript; and decision to submit the manuscript for publication.

## Conflict of Interest

The authors declare that the research was conducted in the absence of any commercial or financial relationships that could be construed as a potential conflict of interest.

## Supplementary Material

Supplementary Figure 1 Time- and concentration-dependent changes in proliferating and total oligodendrocyte lineage cell number. Pure OPCs were isolated from glia mixed culture at DIV 10 and seeded on PDL-coated coverslips, and incubated with gintonin of the indicated concentrations from DIV1 and fixed at the indicated time point for staining of Ki67 (proliferation marker) or DAPI (nucleic marker). Proliferating **(A**, **C**, **E**, **H)** and total cells **(B**, **D**, **F**, **I)** were automatically counted by image J. Bars represent mean ± S.E.M. **p*< 0.05, One way ANOVA, Tukey post-test, N = 3.

Supplementary Figure 2 No effects of Ki16425 on cell survival in physiological condition of OPC cultures. **(A** and **B)** Oligodendrocyte precursor cell (OPC) cultures were incubated with control or Ki16425 as indicated during DIV1–3 in proliferation medium. Scale bar, 100 µm. **(C)** The number of DAPI-positive cells were counted. Bars represent mean ± S.E.M.

Supplementary Figure 3 Expression analysis of myelin protein expression by gintonin and LPA 1/3 inhibitor. Oligodendrocyte precursor cell (OPC) cultures were incubated with gintonin and/or Ki16425 as indicated during DIV1–5. Fresh medium was provided every two days. 7.6 × 10^4^ OPCs per well in the 24-well plate were seeded and collected by 50 µl of 5× sample buffer per well. For Western blot, 9 µl **(A**–**H)** or 3 µl **(I**–**L)** of samples was loaded and immunoblotted with antibodies against MBP (A and B, E and F, I and J). Antibodies on the membrane were stripped and reblotted with antibodies against beta actin **(C** and **D**, **G** and **H**,** K** and **L)**.

Supplementary figure 4 Phosphorylation analysis of ERK by long term treatment of gintonin. Same sample preparation as in **Supplementary Figure 3** was performed. Different antibodies were used for immunoblot; antibodies against phospho ERK **(A** and **B**,** E** and **F**,** I** and **J)** and ERK **(C** and **D**, **G** and **H**, **K** and **L**) were used.

Supplementary Figure 5 Gintonin does not induce dose-dependence changes in the late stage differentiation of oligodendrocytes. **(A**–**C)** For stage-specific differentiation analysis, pure OPC cultures treated with gintonin (1, 10 µg/ml) or DMSO from DIV1 were lively stained at DIV3 with O4 (red) antibodies, then allowed to fix for MBP (green) and DAPI (blue) staining. Scale bar = 100 µm. **(D)** DAPI+ cell number per field by gintonin treatment. **(E)** O4+ area per field by gintonin treatment. **(F)** MBP+ area per field by gintonin. **(G)** O4+ area per DAPI+ cell number (**p*< 0.05). **(H)** MBP+ area per DAPI+ cell number. **(I)** MBP+ area per O4+ area. Bars represent mean ± S.E.M. One way ANOVA, Tukey post-test, N = 3–4.

Supplementary Figure 6 The expression of ER stress marker BiP is induced by tunicamycin. **(A)** In our system, the expression of ER stress marker BiP was confirmed by tunicamycin treatment on OPC cultures at DIV4. Western blot image shows results from two different primary cultures (#1, #2). ERK is shown for loading control.

Supplementary Figure 7 Modulation of gintonin on protein expression under *in vitro* chronic ER stress. **(A**–**D)** Oligodendrocyte precursor cell (OPC) cultures were incubated with gintonin and/or Ki16425 as indicated during DIV1–5 under chronic ER stress induced by tunicamycin. Fresh medium was provided every two days. Protein expression was investigated by ß-actin. Three independent experiments were performed **(A**–**C)** and summarized in one graph **(D)**. Bars represent mean ± S.E.M.

## References

[B1] Allard J. Barron S. Diaz J. Lubetzki C. Zalc B. Schwartz J. C. (1998). A rat G protein-coupled receptor selectively expressed in myelin-forming cells. Eur. J. Neurosci. 10, 1045–1053. 10.1046/j.1460-9568.1998.00117.x 9753172

[B2] Bergles D. E. Richardson W. D. (2016). Oligodendrocyte development and plasticity. Cold Spring Harb. Perspect. Biol. 8, a020453, 1–27. 10.1101/cshperspect.a020453 PMC4743079 26492571

[B3] Boyd A. Zhang H. Williams A. (2013). Insufficient OPC migration into demyelinated lesions is a cause of poor remyelination in MS and mouse models. Acta Neuropathol. 125, 841–859. 10.1007/s00401-013-1112-y 23595275 PMC3661931

[B4] Bradl M. Lassmann H. (2010). Oligodendrocytes: biology and pathology. Acta Neuropathol. 119, 37–53. 10.1007/s00401-009-0601-5 19847447 PMC2799635

[B5] Cahoy J. D. Emery B. Kaushal A. Foo L. C. Zamanian J. L. Christopherson K. S. (2008). A transcriptome database for astrocytes, neurons, and oligodendrocytes: a new resource for understanding brain development and function. J. Neurosci. 28, 264–278. 10.1523/JNEUROSCI.4178-07.2008 18171944 PMC6671143

[B6] Chang A. Nishiyama A. Peterson J. Prineas J. Trapp B. D. (2000). NG2-positive oligodendrocyte progenitor cells in adult human brain and multiple sclerosis lesions. J. Neurosci. 20, 6404–6412. 10.1523/JNEUROSCI.20-17-06404.2000 10964946 PMC6772992

[B7] Choi J. H. Jang M. Oh S. Nah S. Y. Cho I. H. (2018). Multi-target protective effects of gintonin in 1-methyl-4-phenyl-1,2,3,6-tetrahydropyridine-mediated model of parkinson’s disease *via* lysophosphatidic acid receptors. Front Pharmacol. 9, 515. 10.3389/fphar.2018.00515 29875659 PMC5974039

[B8] Choi S. H. Kim H. J. Cho H. J. Park S. D. Lee N. E. Hwang S. H. (2019). Gintonin, a ginseng-derived exogenous lysophosphatidic acid receptor ligand, protects astrocytes from hypoxic and re-oxygenation stresses through stimulation of astrocytic glycogenolysis. Mol. Neurobiol. 56, 3280–3294. 10.1007/s12035-018-1308-1 30117105

[B9] Clayton B. L. L. Popko B. (2016). Endoplasmic reticulum stress and the unfolded protein response in disorders of myelinating glia. Brain Res. 1648, 594–602. 10.1016/j.brainres.2016.03.046 27055915 PMC5036997

[B10] Elbaz B. Popko B. (2019). Molecular control of oligodendrocyte development. Trends Neurosci. 42, 263–277. 10.1016/j.tins.2019.01.002 30770136 PMC7397568

[B11] Emery B. Lu Q. R. (2015). Transcriptional and epigenetic regulation of oligodendrocyte development and myelination in the central nervous system. Cold Spring Harb. Perspect. Biol. 7, a020461. 10.1101/cshperspect.a020461 26134004 PMC4563712

[B12] Finzsch M. Stolt C. C. Lommes P. Wegner M. (2008). Sox9 and Sox10 influence survival and migration of oligodendrocyte precursors in the spinal cord by regulating PDGF receptor alpha expression. Development 135, 637–646. 10.1242/dev.010454 18184726

[B13] Guardiola-Diaz H. M. Ishii A. Bansal R. (2012). Erk1/2 MAPK and mTOR signaling sequentially regulates progression through distinct stages of oligodendrocyte differentiation. Glia 60, 476–486. 10.1002/glia.22281 22144101 PMC3265651

[B14] Harding H. P. Novoa I. Zhang Y. Zeng H. Wek R. Schapira M. (2000). Regulated translation initiation controls stress-induced gene expression in mammalian cells. Mol. Cell 6, 1099–1108. 10.1016/S1097-2765(00)00108-8 11106749

[B15] Hughes E. G. Appel B. (2016). The cell biology of CNS myelination. Curr. Opin. Neurobiol. 39, 93–100. 10.1016/j.conb.2016.04.013 27152449 PMC4987163

[B16] Hwang S. H. Lee B. H. Choi S. H. Kim H. J. Won K. J. Lee H. M. (2016). Effects of gintonin on the proliferation, migration, and tube formation of human umbilical-vein endothelial cells: involvement of lysophosphatidic-acid receptors and vascular-endothelial-growth-factor signaling. J. Ginseng Res. 40, 325–333. 10.1016/j.jgr.2015.10.002 27746684 PMC5052429

[B17] Hwang S. H. Shin T. J. Choi S. H. Cho H. J. Lee B. H. Pyo M. K. (2012). Gintonin, newly identified compounds from ginseng, is novel lysophosphatidic acids-protein complexes and activates G protein-coupled lysophosphatidic acid receptors with high affinity. Mol. Cells 33, 151–162. 10.1007/s10059-012-2216-z 22286231 PMC3887723

[B18] Levine J. M. Reynolds R. Fawcett J. W. (2001). The oligodendrocyte precursor cell in health and disease. Trends Neurosci. 24, 39–47. 10.1016/S0166-2236(00)01691-X 11163886

[B19] Lin W. Popko B. (2009). Endoplasmic reticulum stress in disorders of myelinating cells. Nat. Neurosci. 12, 379–385. 10.1038/nn.2273 19287390 PMC2697061

[B20] Lucchinetti C. Bruck W. Parisi J. Scheithauer B. Rodriguez M. Lassmann H. (1999). A quantitative analysis of oligodendrocytes in multiple sclerosis lesions. A study of 113 cases. Brain 122 (Pt 12), 2279–2295. 10.1093/brain/122.12.2279 10581222

[B21] Mi Kyung Pyo S.-H. C. Hwang S. H. Shin T.-J. Lee Sang B.-H. Mok Lee Y.-H. L. Kim D.-H. (2011). , Novel glycolipoproteins from ginseng. J. Ginseng Res. 35, 92–103. 10.5142/jgr.2011.35.1.092

[B22] Nakajima S. Gotoh M. Fukasawa K. Murofushi H. Murakami Murofushi K. (2018). 2-O-Carba-oleoyl cyclic phosphatidic acid induces glial proliferation through the activation of lysophosphatidic acid receptor. Brain Res. 1681, 44–51. 10.1016/j.brainres.2017.12.031 29278716

[B23] Tsaytler P. Harding H. P. Ron D. Bertolotti A. (2011). Selective inhibition of a regulatory subunit of protein phosphatase 1 restores proteostasis. Science 332, 91–94. 10.1126/science.1201396 21385720

[B24] Weiner J. A. Hecht J. H. Chun J. (1998). Lysophosphatidic acid receptor gene vzg-1/lpA1/edg-2 is expressed by mature oligodendrocytes during myelination in the postnatal murine brain. J. Comp Neurol. 398, 587–598. 10.1002/(SICI)1096-9861(19980907)398:4<587::AID-CNE10>3.0.CO;2-5 9717712

[B25] Wheeler N. A. Lister J. A. Fuss B. (2015). The autotaxin-lysophosphatidic acid axis modulates histone acetylation and gene expression during oligodendrocyte differentiation. J. Neurosci. 35, 11399–11414. 10.1523/JNEUROSCI.0345-15.2015 26269646 PMC4532767

[B26] Yang H. J. Vainshtein A. Maik-Rachline G. Peles E. (2016). G protein-coupled receptor 37 is a negative regulator of oligodendrocyte differentiation and myelination. Nat. Commun. 7, 10884. 10.1038/ncomms10884 26961174 PMC4792952

[B27] Yu N. Lariosa-Willingham K. D. Lin F. F. Webb M. Rao T. S. (2004). Characterization of lysophosphatidic acid and sphingosine-1-phosphate-mediated signal transduction in rat cortical oligodendrocytes. Glia 45, 17–27. 10.1002/glia.10297 14648542

