# Supplementary figures and images for: Gintonin Enhances Proliferation, Late Stage Differentiation, and Cell Survival From Endoplasmic Reticulum Stress of Oligodendrocyte Lineage Cells
Source: Front Pharmacol. 2019 Oct 8;10:1211. doi: 10.3389/fphar.2019.01211 (PMC6797839; doi:10.3389/fphar.2019.01211)

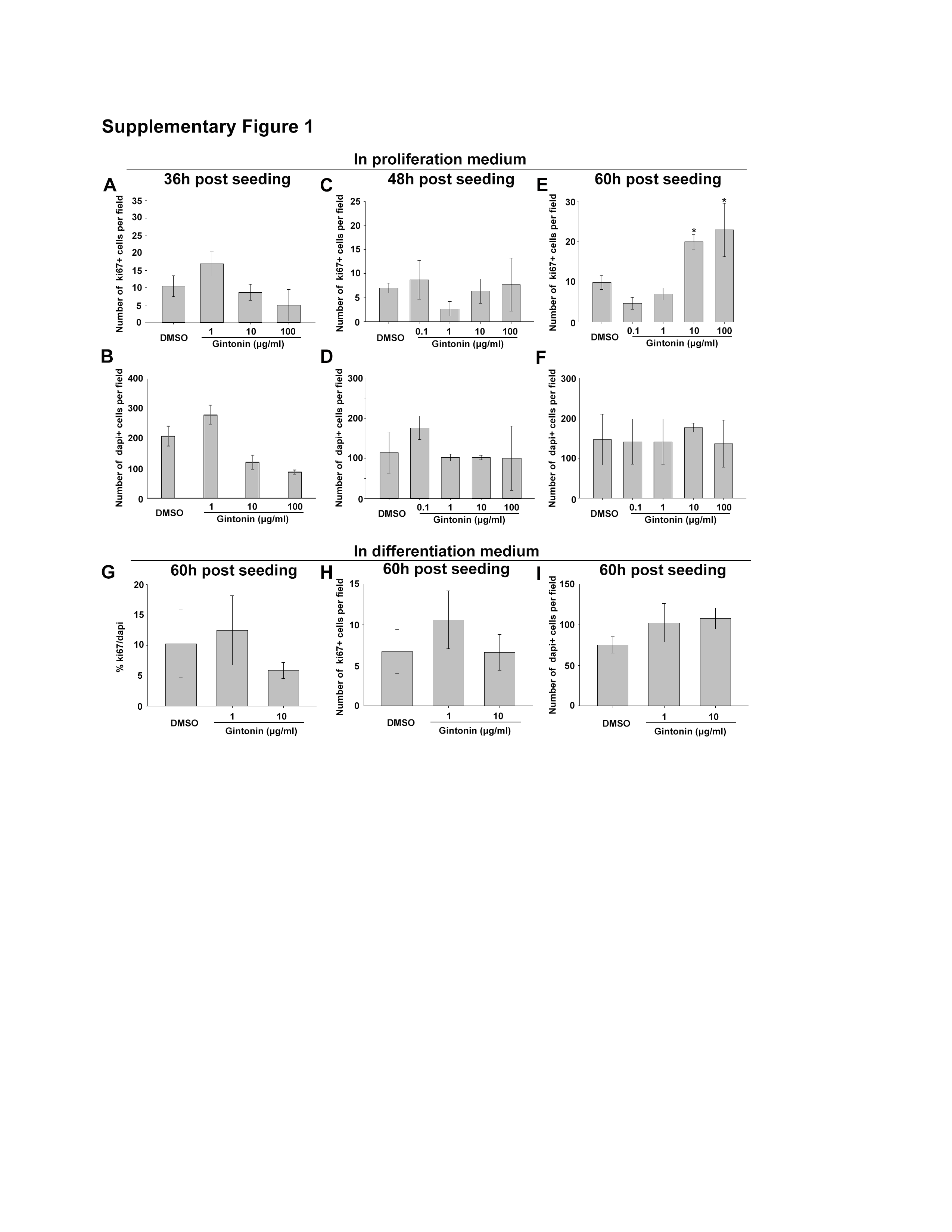

Supplement: Supplementary Figure 1 — Time- and concentration-dependent changes in proliferating and total oligodendrocyte lineage cell number. Pure OPCs were isolated from glia mixed culture at DIV 10 and seeded on PDL-coated coverslips, and incubated with gintonin of the indicated concentrations from DIV1 and fixed at the indicated time point for staining of Ki67 (proliferation marker) or DAPI (nucleic marker). Proliferating (A, C, E, H) and total cells (B, D, F, I) were automatically counted by image J. Bars represent mean ± S.E.M. *p< 0.05, One way ANOVA, Tukey post-test, N = 3. [file Image_1.tif]

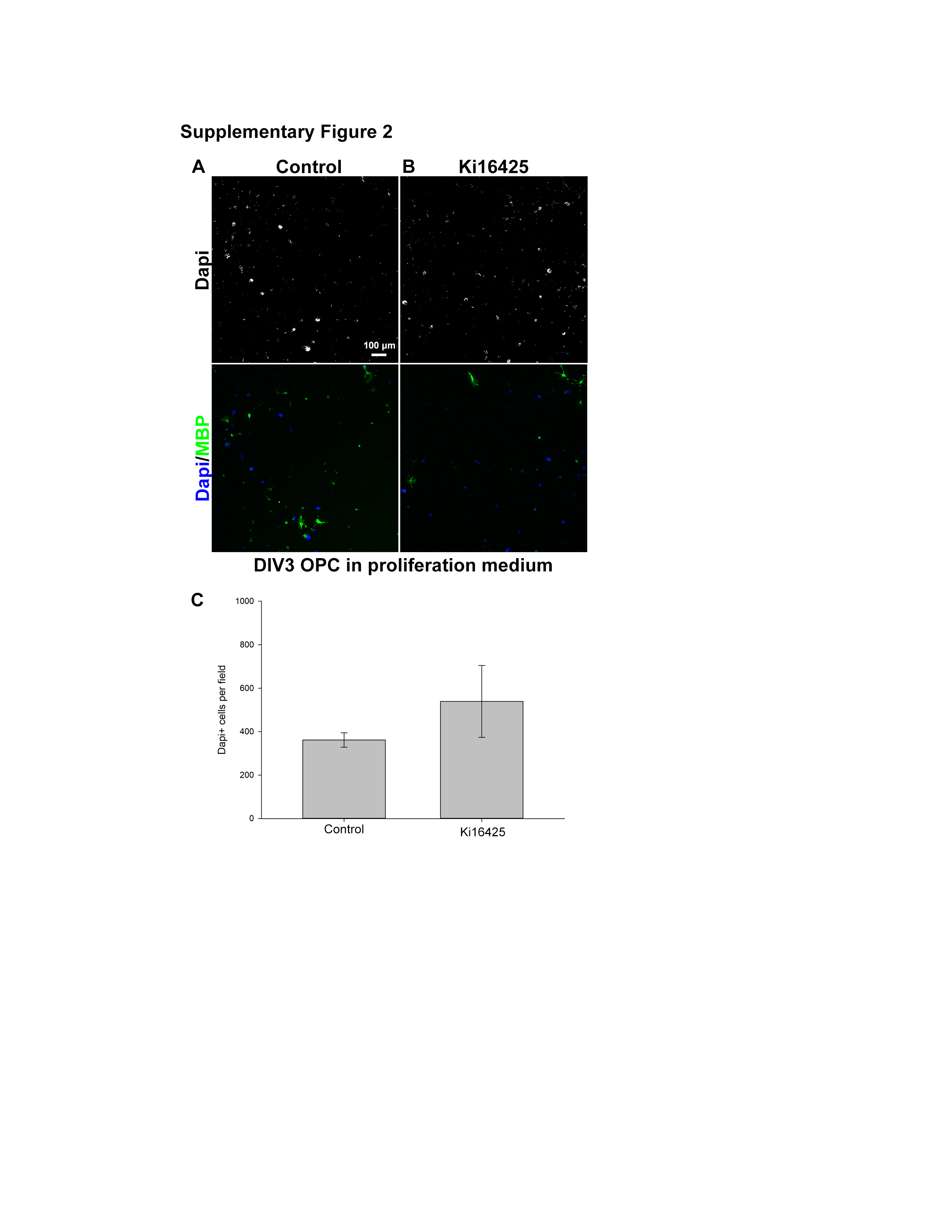

Supplement: Supplementary Figure 2 — No effects of Ki16425 on cell survival in physiological condition of OPC cultures. (A and B) Oligodendrocyte precursor cell (OPC) cultures were incubated with control or Ki16425 as indicated during DIV1–3 in proliferation medium. Scale bar, 100 µm. (C) The number of DAPI-positive cells were counted. Bars represent mean ± S.E.M. [file Image_2.tif]

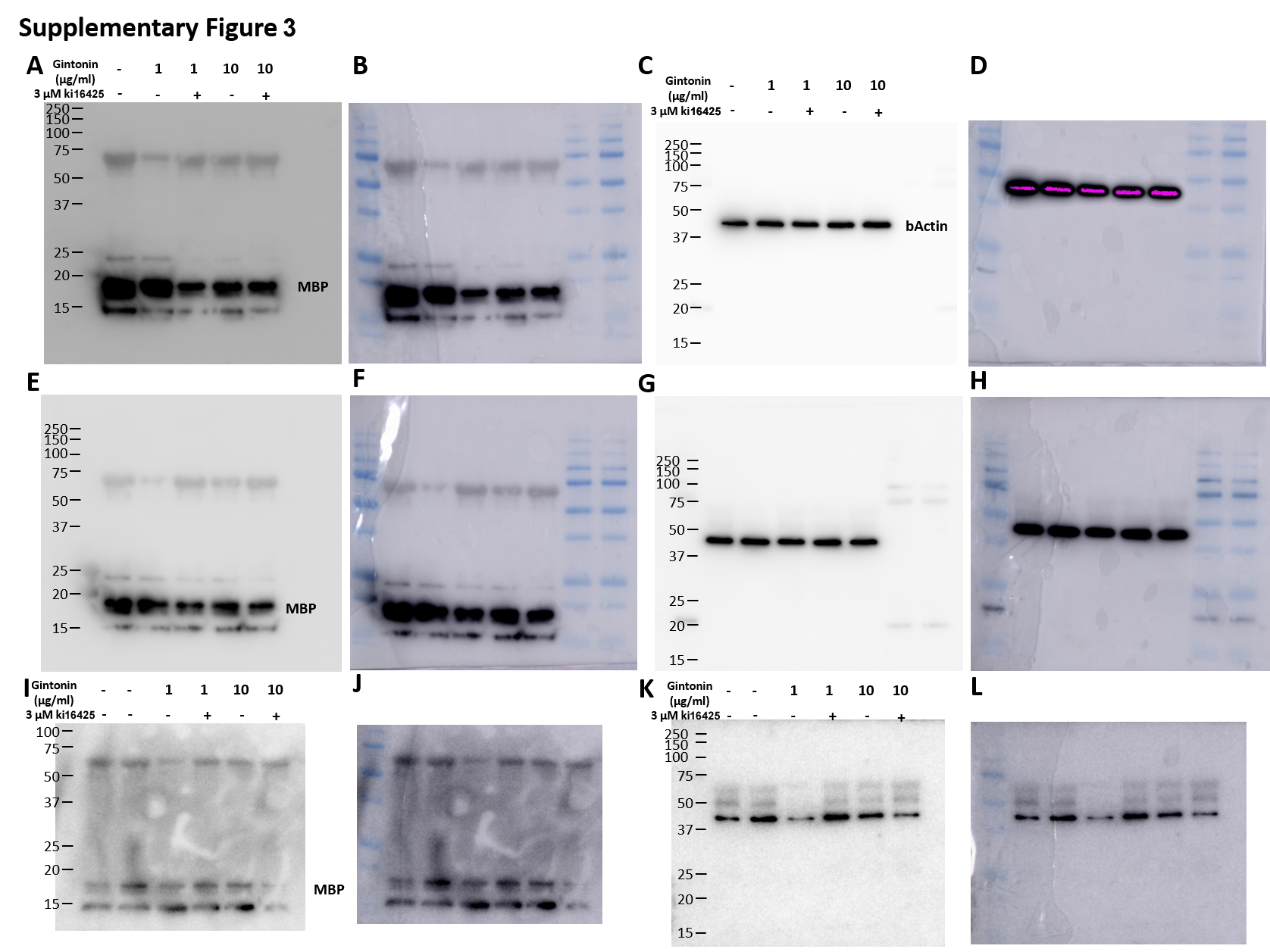

Supplement: Supplementary Figure 3 — Expression analysis of myelin protein expression by gintonin and LPA 1/3 inhibitor. Oligodendrocyte precursor cell (OPC) cultures were incubated with gintonin and/or Ki16425 as indicated during DIV1–5. Fresh medium was provided every two days. 7.6 × 10^4^ OPCs per well in the 24-well plate were seeded and collected by 50 µl of 5× sample buffer per well. For Western blot, 9 µl (A–H) or 3 µl (I–L) of samples was loaded and immunoblotted with antibodies against MBP (A and B, E and F, I and J). Antibodies on the membrane were stripped and reblotted with antibodies against beta actin (C and D, G and H, K and L). [file Image_3.tif]

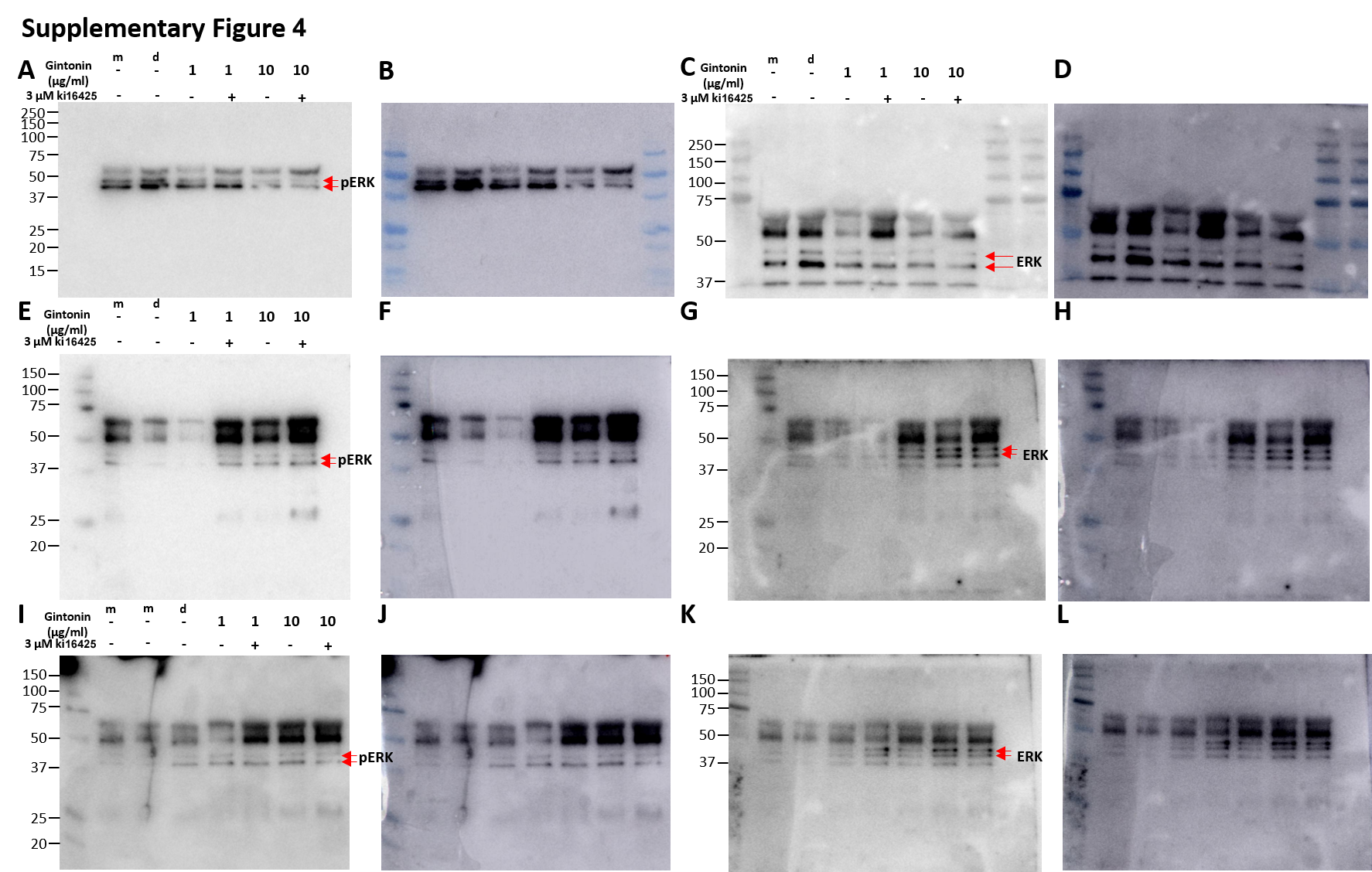

Supplement: Supplementary figure 4 — Phosphorylation analysis of ERK by long term treatment of gintonin. Same sample preparation as in Supplementary Figure 3 was performed. Different antibodies were used for immunoblot; antibodies against phospho ERK (A and B, E and F, I and J) and ERK (C and D, G and H, K and L) were used. [file Image_4.tif]

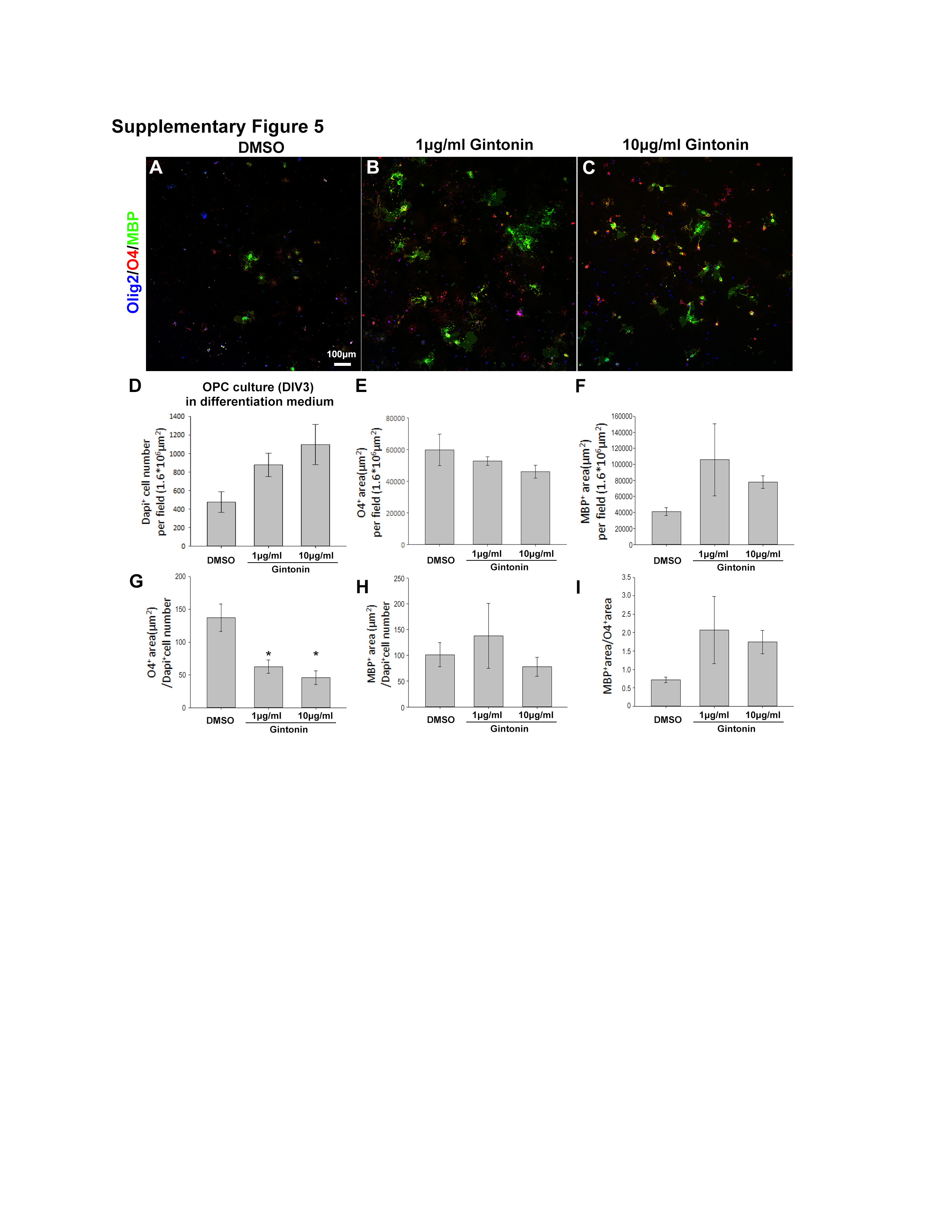

Supplement: Supplementary Figure 5 — Gintonin does not induce dose-dependence changes in the late stage differentiation of oligodendrocytes. (A–C) For stage-specific differentiation analysis, pure OPC cultures treated with gintonin (1, 10 µg/ml) or DMSO from DIV1 were lively stained at DIV3 with O4 (red) antibodies, then allowed to fix for MBP (green) and DAPI (blue) staining. Scale bar = 100 µm. (D) DAPI+ cell number per field by gintonin treatment. (E) O4+ area per field by gintonin treatment. (F) MBP+ area per field by gintonin. (G) O4+ area per DAPI+ cell number (*p< 0.05). (H) MBP+ area per DAPI+ cell number. (I) MBP+ area per O4+ area. Bars represent mean ± S.E.M. One way ANOVA, Tukey post-test, N = 3–4. [file Image_5.tif]

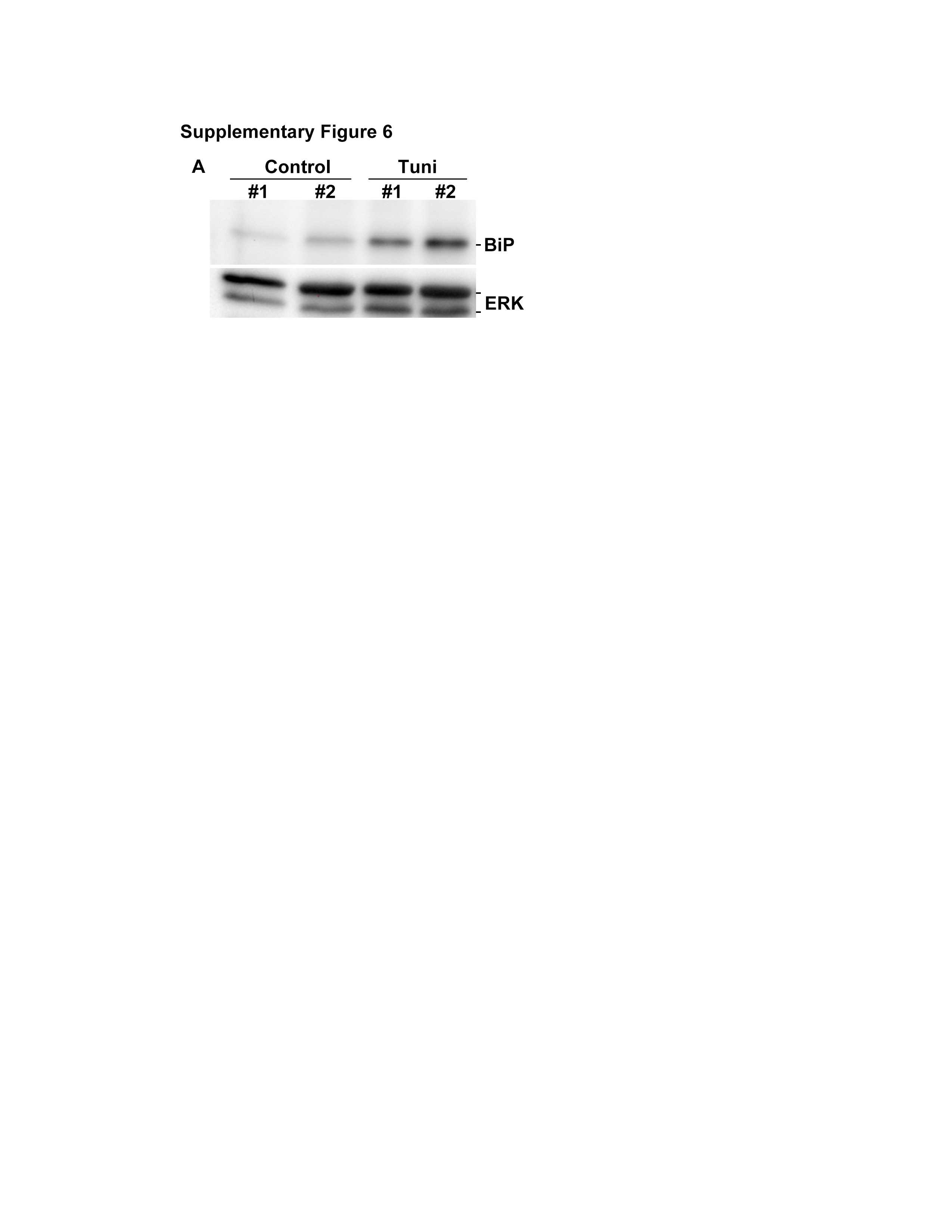

Supplement: Supplementary Figure 6 — The expression of ER stress marker BiP is induced by tunicamycin. (A) In our system, the expression of ER stress marker BiP was confirmed by tunicamycin treatment on OPC cultures at DIV4. Western blot image shows results from two different primary cultures (#1, #2). ERK is shown for loading control. [file Image_6.tif]

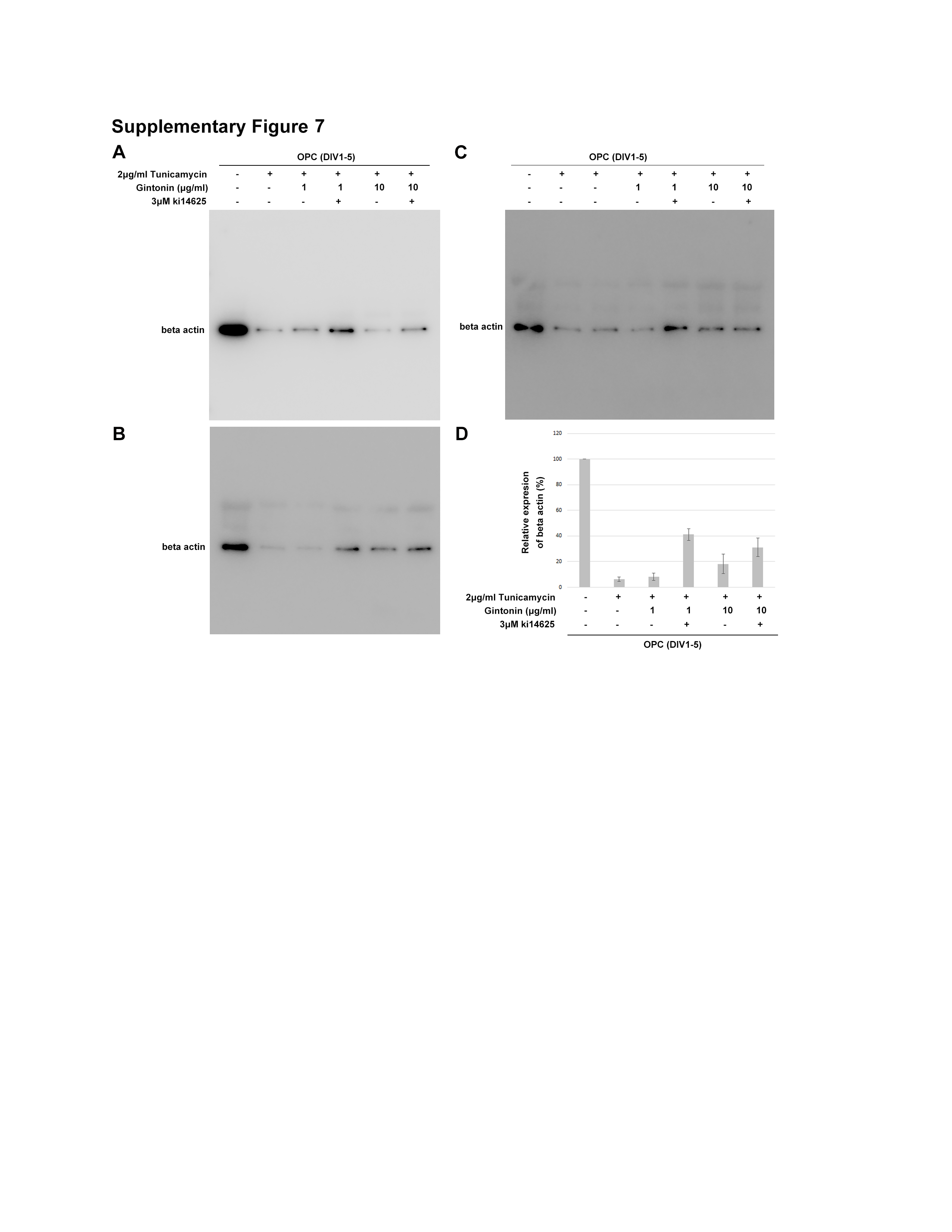

Supplement: Supplementary Figure 7 — Modulation of gintonin on protein expression under in vitro chronic ER stress. (A–D) Oligodendrocyte precursor cell (OPC) cultures were incubated with gintonin and/or Ki16425 as indicated during DIV1–5 under chronic ER stress induced by tunicamycin. Fresh medium was provided every two days. Protein expression was investigated by ß-actin. Three independent experiments were performed (A–C) and summarized in one graph (D). Bars represent mean ± S.E.M. [file Image_7.tif]
